# A Polyethylene Base Moisture Activating Oxygen Scavenging Film Co-Extruded with Tea Polyphenols-β-Cyclodextrin Inclusion Complex

**DOI:** 10.3390/ma13173857

**Published:** 2020-09-01

**Authors:** Liao Pan, Meiying Zhang, Lixin Lu, Bingxian Ou, Xi Chen

**Affiliations:** 1 Department of Packaging Engineering, Jiangnan University, Wuxi 214122, China; breath860101@aliyun.com (L.P.); 15061889036@163.com (M.Z.); 8201901012@jiangnan.edu.cn (X.C.); 2 Jiangsu Key Laboratory of Advanced Food Manufacturing Equipment and Technology, Wuxi 214122, China; 3 National Graphene Products Quality Supervision and Inspection Center (Jiangsu), Special Equipment Safety Supervision Inspection Institute of Jiangsu Province, Wuxi 214174, China

**Keywords:** moisture activating, oxygen scavenging film, tea polyphenols, β-cyclodextrin, low-density polyethylene (LDPE)

## Abstract

Antioxidant packaging is an effective method to protect oxygen-sensitive food from oxidation. In order to concurrently obtain a storage stability and excellent oxygen scavenging of antioxidant film for the high moisture food, a moisture activating oxygen scavenging film was prepared by using tea polyphenols as the oxygen scavenger. The moisture activating function was achieved by introducing the β-cyclodextrin embedding technology, and the tea polyphenols–β-cyclodextrin inclusion complex was co-extruded with low-density polyethylene (LDPE) to improve the storage stability. The results indicate that the tea polyphenols is well embedded by β-cyclodextrin according to the Fourier transform infrared spectra (FT-IR), and a relatively homogeneous dispersion of oxygen scavenger is observed while the oxygen scavenger content is less than 5%. The oxygen scavenging increases with the increase of oxygen scavenger from 1% to 5%, and a maximal oxygen absorption of 0.0150 mol/m^2^ is exhibited at oxygen scavenger content value of 5%. Then, the oxygen scavenging significantly decrease under the oxygen scavenger content of 7% and 10%. Moreover, the oxygen scavenging amount sharply increase after steeping in water or storage in extremely high humidity of RH 84% while the oxygen scavenging is restrained under RH 32–75%, indicating that the moisture activating oxygen scavenging is functioning. The oxygen scavenging is obvious restrained under low temperature of 4 °C while the oxygen scavenging is activated at 23 °C and 50 °C with similar oxygen scavenging amount. Besides, both of the tensile and heat-sealing strength deteriorative with the increase of oxygen scavenger content, while they are acceptable at oxygen scavenger content of 5%. Finally, the prepared oxygen scavenging film was used for packaging orange juice and received a good antioxidant effect. Thus, the acquired moisture activating oxygen scavenging film has a good stability under regular storage condition, and shows a potentially application for oxygen-sensitive food with high moisture content.

## 1. Introduction

Oxidation is one of the major reasons of food deterioration and flavor altering [1]. The traditional way is using an oxygen barrier film to prevent from the oxygen permeation. But the residual oxygen inside packages will also bring an oxidation. Antioxidant packaging provides an effective method to thoroughly eliminate the residual oxygen, and protects oxygen-sensitive food from oxidation [2]. A common way is releasing active substances into food system to scavenging free radical or interdicting peroxide. Most of the antioxidant will introduce negative flavor which is unacceptable in some situations [3]. An alternative method is blending oxygen scavenger in packaging materials, and the oxygen in top space of packaging can be absorbed [4,5,6]. However, the oxygen scavenging process not only exists in the storage period after packaging, but also occurs before packaging. This premature oxygen absorption will overdraft the oxygen scavenger, and the oxygen scavenger may be exhausted before packaging or during the shelf life of foods. Thus, the academic community shown a renewed interest in exploiting stimuli-activating films to fit different requests of before and after packaging.

The stimuli-activating films can rapidly switch from passivated state to activated state under a specific stimulation, and the microstructure of film can respond to this external stimulation to drastically change the film performances. Temperature is the most common activating factor used in stimuli-activating films. Most of the active substance release process can be accelerated under a high temperature due to the mechanism of Fick diffusion, and numerous temperature-activating materials were developed based on this mechanism [7,8]. Temperature also affects the crystal process which leads to a sharply change of free volume and permeability. Chen prepared a double-switch temperature-sensitive controlled release antioxidant film by introducing a temperature sensitive polyurethane (TSPU) which has a significant phase change in a certain temperature range [9]. This film showed two significant release acceleration under the two phase change temperatures. pH value is another general activating factor for stimuli-activating films. pH value change causes a charge change, even a charge polarity turning, which will also affect the permeability of films, furthermore accelerate or restrain the release process [10,11,12]. Ultraviolet (UVA) [13], near-infrared (NIR) [14] even natural daylight [15] also has been used as an activating factor to control the diffusion and reaction rate of active substances. However, moisture as an effective and convenient factor, except the aforementioned activating factors, is scarce in previous researches. The microstructure of hydrophilic materials is significantly affected by moisture, and this character brings an expectable application prospect of moisture activating films for oxygen-sensitive food with high moisture content.

Tea polyphenol is a widely applicated antioxidant with a general term of multi-phenolic compounds in tea [16] The current researches mainly focus on the antioxygenation of tea polyphenols [17,18,19], and tea polyphenols has been used in some products in market as an antioxidant [20]. Nevertheless, there has been no research on tea polyphenol as an oxygen scavenger in packaging system to the author’s knowledge. Previous studies showed that tea polyphenols could react with oxygen due to the active hydroxyl group in its molecule structure [21]. These researches provide a theoretical basis for the application of tea polyphenols as an oxygen scavenger. However, the polyhydroxy structure of tea polyphenol has a high reaction activity and easily deteriorative due to the temperature, ultraviolet light and other environmental factors [22]. Furthermore, the terrible thermal stability of tea polyphenols leads to that tea polyphenols cannot fits the high temperature during plastic forming [23]. Thus, the β-cyclodextrin embedded technique was introduced to improve the stability of tea polyphenols. β-cyclodextrin is a cyclic compound composed by 7 glucose molecules in the form of β-1,4-glycosidic bond. The main structure of β-cyclodextrin is a hollow cylinder which has the property of “internal hydrophobic and external hydrophilic”. This hollow cylinder can envelop one target molecule into a stable inclusion complex [24,25]. On one hand, this inclusion complex effectively improves the thermal stability of tea polyphenols [26], and also restrains the oxygen scavenging of tea polyphenols in dry environment [27]. On the other hand, the oxygen scavenging of tea polyphenols can be activated while the external hydrophilic structure of β-cyclodextrin is destroyed under high humidity. This moisture sensibility provides a definitely possible for preparing a moisture activating oxygen scavenging film.

The aim of this study is to prepare polyethylene base moisture activating oxygen scavenging film co-extruded with tea polyphenols-β-cyclodextrin inclusion complex. Then investigate the microstructure, analyze the influence of the oxygen scavenger content, humidity, activating condition, temperature on the oxygen scavenging performance and the mechanical properties of the prepared film. Besides, an application example of this novel material is also provided.

## 2. Materials and Methods

### 2.1. Materials

Tea polyphenols (99.23%), purchased from Guangdong Chen Yuan Fine Chemical Co, Ltd (Foshan, China). The tea polyphenols is extracted from green tea by supercritical fluid extraction. β-cyclodextrin, purchased from Aladdin reagent (Shanghai, China) Co., Ltd. Absolute ethanol (AR), purchased from Sinopharm Chemical Reagent Co., Ltd (Shanghai, China). Low-density polyethylene (LDPE) resin (LD100AC), purchased from Exxon Mobil (Shanghai, China) Investment Co., Ltd.

### 2.2. Methods

#### 2.2.1. Preparation of Moisture Activating Oxygen Scavenging Film

β-cyclodextrin (12 g) was dissolved in deionized water, and 6 g tea polyphenols were dissolved in ethanol solution. The tea polyphenols solution was lowly added into the β-cyclodextrin solution and the mixed solution stirred under 500 rpm at 60 °C for 4 h. Placed the mixture under 4 °C for 3 days, and then, white powder was obtained through vacuum pump suction (SHK-III, KETAI, Zhengzhou, China). The adhering solvents were evaporated through vacuum dryer (DZF-6050, JIANGHONG, Shanghai, China) and the tea polyphenols–β-cyclodextrin inclusion complex was prepared as an oxygen scavenger.

According to Table 1, LDPE resin and oxygen scavenger were uniformly mixed in a high-speed mixer (LMX5-VS, LAB TECH, Bangkok, Thailand). The mixed resin pellets were fused, granulated and co-extruded into film through a pelletizer (LTE16-40, LAB TECH, Bangkok, Thailand) and a co-extrusion casting machine (LMCR-300, LAB TECH, Bangkok, Thailand). The related parameters are shown in Table 2. The thickness of the prepared film was 103 ± 9 μm controlled by 5 micrometers (Q/ILBN2-2006CH-1-S, LIULING, Shanghai, China). The obtained film was stored in a vacuum desiccator.

#### 2.2.2. Fourier Transform Infrared (FT-IR) Spectroscopy

A FT-IR (Nicolet460, THERMO FISHER SCIENTIFIC, Waltham, MA, USA) was used to characterize the structure of tea polyphenols-β-cyclodextrin inclusion complex under the scanning range of 600–4000 cm^–1^.

#### 2.2.3. Dispersion Characterization

The particle distribution of oxygen scavenger in film is measured by using equivalent projected circular area diameter (EQPC) method [28]. The dispersion of oxygen scavenger was also characterized using a microscope (BM103CE, BIMU, Shanghai, China).

#### 2.2.4. Oxygen Scavenging Characterization

**Figure 1:**
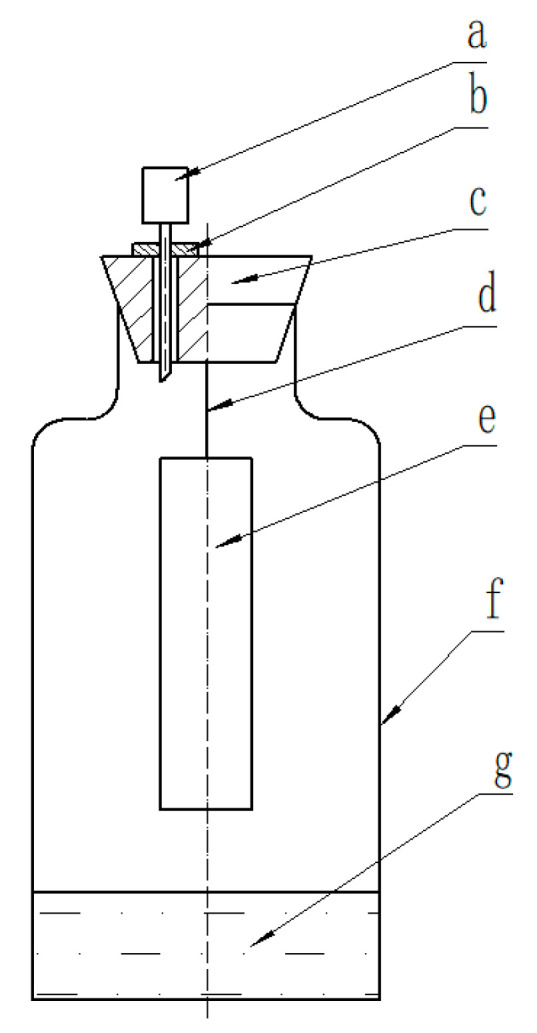
A closed system of oxygen scavenging test. (**a**). Probe of headspace O_2_/CO_2_ analyzer; (**b**). sealing gel; (**c**). plug; (**d**). string; (**e**) sample; (**f**) 100 mL glass bottle; (**g**) saturated solution (only used in the influence of humidity).

As shown in Figure 1, the prepared oxygen scavenging film was cut into pieces of 5 cm × 8 cm to test the oxygen scavenging performance by using a closed system referring to the literature [29]. The oxygen content inside the glass bottle could be measure by a headspace O_2_/CO_2_ analyzer (IIIinois 6600, MOCON, Minneapolis, MN, USA) through a sealing gel which could reseal the plug after puncturing by the probe. Then, the oxygen scavenging capacity of the oxygen scavenging film was calculated through the Equation (1) based on the oxygen content change.

(1) $$OS=\frac{PV}{2ART}\frac{C_{i}-C_{t}}{1-C_{t}}$$

where, OS is the amount of oxygen scavenging/mol/m^2^; P is the standard atmospheric pressure, equal to 1.013 × 10^5^ Pa; V is the volume of glass bottle/m^3^; A is the area of film sample/m^2^; R is the perfect gas constant, equal to 8.314 J/(mol·K); T is the temperature/K; $C_{i}$ and $C_{t}$ are the oxygen content of initial time and real time/%.

##### Influence of Oxygen Scavenger Content

Different oxygen scavenger contents of film sample were hanged and sealed in 100 mL dry glass bottles after activated by water (immersed in deionized water for 5 min) and placed at a temperature of 30 °C controlled by a temperature and humidity chamber (J85-2, KSON, Kaohsiung, Taiwan). The oxygen contents in the glass bottles were periodically measured by headspace O_2_/CO_2_ analyzer, until the oxygen contents were no longer decreased. The oxygen scavenging capacity of the oxygen scavenging film was calculated through Equation (1).

##### Influence of Humidity

The influence of humidity is necessary due to the water sensitivity of the prepared oxygen scavenging film. Film samples with 5% oxygen scavenger were respectively hanged and sealed in a 100 mL glass bottles with different relative humidity (RH) and placed at a temperature of 30 °C. The different relative humidity is achieved by different saturated solution listed in Table 3. The oxygen scavenging capacity was calculated by Equation (1).

##### Influence of Activating Condition

A piece of activated film sample (immersed in deionized water for 5 min) with 5% oxygen scavenger was hanged and sealed in a 100 mL glass bottle. Another film sample which was not activated was also hanged and sealed in a same bottle. The air in both of the bottles is aforehand dehydrated by desiccant. The storage temperature is 30 °C. The oxygen scavenging capacity of these two activating conditions was calculated by Equation (1).

##### Influence of Temperature

Film samples with 5% oxygen scavenger were hanged and sealed in a 100 mL dry glass bottles after steeped by water, and placed in different temperatures (4 °C, 23 °C and 50 °C) balancing for 15 days. Then, these film samples were activated by water and then also placed in the corresponding temperatures to measure the oxygen contents inside the bottles. The oxygen scavenging capacity was calculated by Equation (1).

#### 2.2.5. Tensile Properties Test

The tensile strength and elongation of the prepared films were determined using a tension test machine (LRX PLUS 5kN, LLOYD, West Sussex, UK). Sample size is 150 × 15 mm^2^, drawing speed is 200 mm/min.

#### 2.2.6. Heat-Sealing Strength Test

The prepared films were heat-sealed by a heat-sealing machine (SL-2, THWING-ALBERT, West Berlin, NJ, USA) under temperature of 130 °C and pressure of 0.35 MPa for 0.7 s. The heat-sealing strength was also determined using the aforementioned tension test machine. Sample size is 150 × 15 mm^2^, drawing speed is 200 mm/min.

#### 2.2.7. Application

Orange juice is rich in vitamin C (VC) which is easily oxidized during storage. Thus, the prepared oxygen scavenging film was used to package orange juice in this study. Orange juice was squeeze by using a juicer (JYZ-E25, JOYOUNG, Hangzhou, China). The prepared film with 5% oxygen scavenger was cut and sealed into a pouch with the size of 120 × 140 mm^2^. The LDPE film without oxygen scavenger was also cut and sealed into a pouch with the size of 120 × 140 mm^2^ as a control group. Orange juice (40 mL) was respectively filled in the two pouches. The samples were placed in the temperature of 30 °C and RH 50%. VC content and brown stain were tested every 5 days.

VC content was measured by 2, 6-dichloroindophenol titration according to Chinese standard of GB/T 6195-1986 [31].

Brown stain was tested by an ultraviolet and visible spectrophotometer (UV-1800, UNICO, Shanghai, China) [32]. Orange juice sample (10 mL) was centrifuged by a centrifugal machine (RJ-TDL-50A, RUIJIANG, Wuxi, China) under 2400 rpm for 150 min. The supernatant liquid was attenuated in 10 mL 95% ethanol solution and percolated by filter paper. The absorbance under 420 nm was identified as the brown stain.

## 3. Results and Discussion

### 3.1. FT-IR Spectroscopy

**Figure 2:**
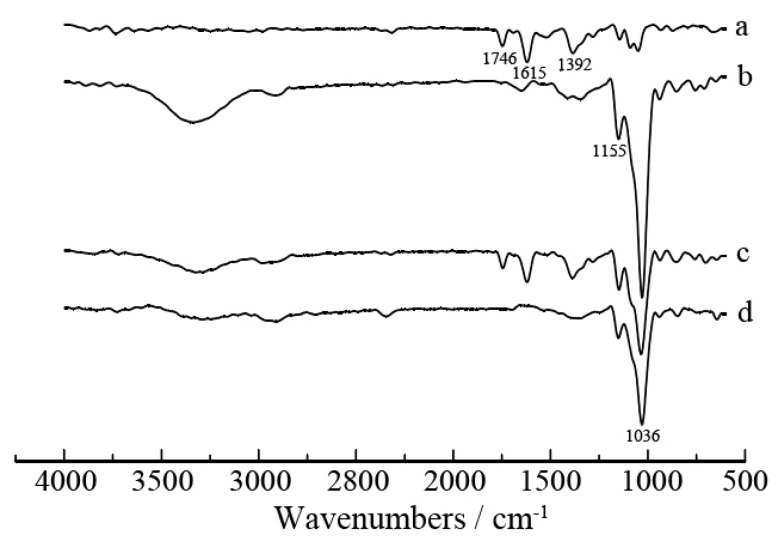
FT-IR spectrogram of tea polyphenols, β-cyclodextrin, tea polyphenols/β-cyclodextrin mixture and tea polyphenols-β-cyclodextrin inclusion complex. (**a**) Tea polyphenols; (**b**) β-cyclodextrin; (**c**) tea polyphenols/β-cyclodextrin mixture; (**d**) tea polyphenols-β-cyclodextrin inclusion complex.

Fourier transform infrared spectra (FT-IR) of tea polyphenols, β-cyclodextrin, tea polyphenols/β-cyclodextrin mixture and tea polyphenols-β-cyclodextrin inclusion complex are depicted in Figure 2. For tea polyphenols, the characteristic absorption peaks are mainly at 1746, 1615 and 1392 cm^–1^. The absorption bands of β-cyclodextrin are at 1155 and 1036 cm^–1^. With the mixture of tea polyphenols and β-cyclodextrin, both of the absorption peaks of tea polyphenols and β-cyclodextrin are existing at 1746, 1615, 1392, 1155 and 1036 cm^–1^. However, the absorption bands of tea polyphenols-β-cyclodextrin inclusion complex are only at 1155 and 1036 cm^–1^ which are the characteristic absorption peaks of β-cyclodextrin. This phenomenon indicates that the tea polyphenols has been included in the hydrophobic cavity of β-cyclodextrin due to the complexation of tea polyphenols with β-cyclodextrin.

### 3.2. Dispersion

**Figure 3:**
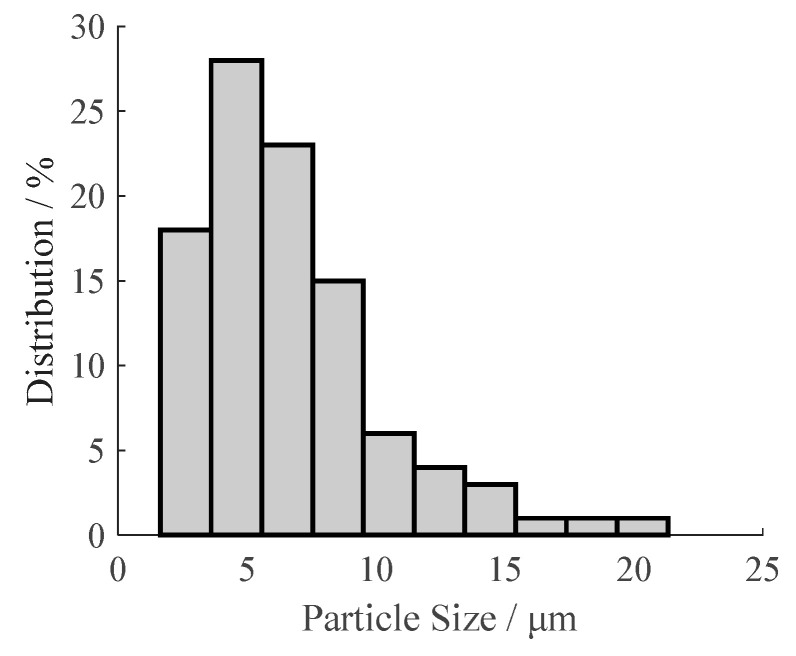
Particle distribution of the prepared oxygen scavenger in film.

**Figure 4:**
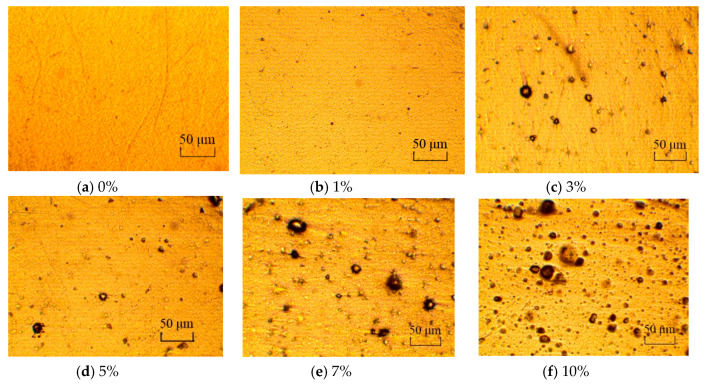
The oxygen scavenger dispersion under different content. (**a**) 0%; (**b**) 1%; (**c**) 3%; (**d**) 5%; (**e**) 7%; (**f**) 10%.

Figure 3 shows the particle distribution of the prepared oxygen scavenger in the prepared film, and the average particle size is 6.58 μm. Figure 4 is the micrographs of the prepared films with different oxygen scavenger contents (0%, 1%, 3%, 5%, 7%, 10%). Micrographs a–d show a relatively homogeneous dispersion of oxygen scavenger, without obvious aggregation and bubble while the oxygen scavenger content is less than 5%. However, it can be observed from Figure 4e and f that the dispersion becomes heterogeneous with the oxygen scavenger content increasing from 7 to 10%. This is chiefly because the hydrophobic LDPE is immiscible with the hygrophilous β-cyclodextrin shell. This immiscibility leads to a more significant inter-attraction among the oxygen scavenger particles with the oxygen scavenger content increasing, and finally causes the aggregation and bubble in the films. The heterogeneous dispersion will further affect the oxygen scavenging performance and tensile properties of the prepared film.

### 3.3. Oxygen Scavenging Performance

#### 3.3.1. Influence of Oxygen Scavenger Content

**Figure 5:**
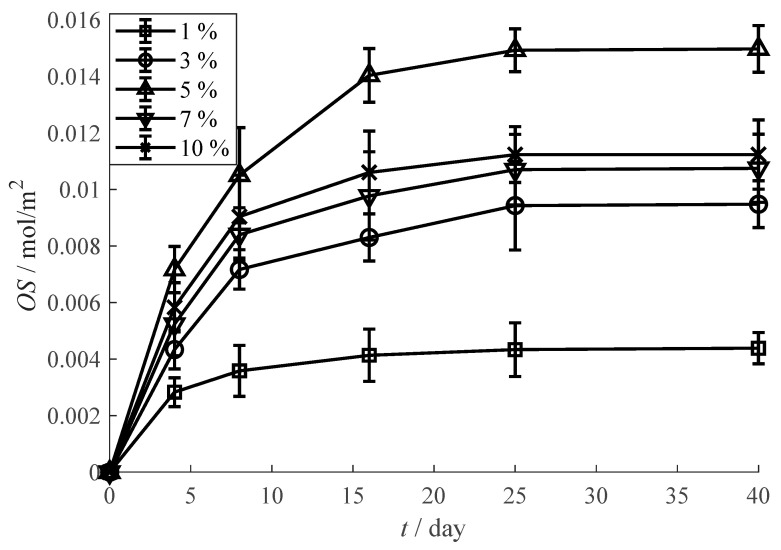
Influence of oxygen scavenger content on the oxygen scavenging performance.

Figure 5 shows the *OS* values of the oxygen scavenging film with different oxygen scavenger contents. The *OS* values at oxygen scavenger content of 1%, 3% and 5% are 0.0044 mol/m^2^, 0.0095 mol/m^2^ and 0.0150 mol/m^2^ respectively. This increase indicates that an obvious improvement of oxygen scavenging performance is obtained while the oxygen scavenger content increases from 1% to 5%. However, the *OS* values at oxygen scavenger content of 7% and 10% respectively decrease to 0.0107 mol/m^2^ and 0.0112 mol/m^2^. This weakening, on one hand, is caused the heterogeneous dispersion of oxygen scavenger under high content. On the other hand, a pro-oxidative character which has been observed in previous studies [33,34] is possibly activated under a high content of tea polyphenols.

#### 3.3.2. Influence of Humidity

**Figure 6:**
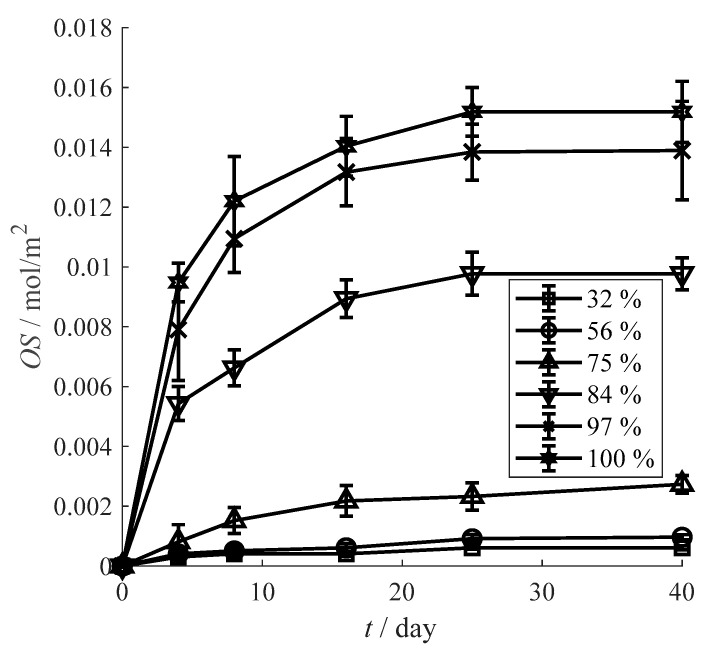
Influence of humidity on the oxygen scavenging performance.

The oxygen scavenging performance of the prepared film is significantly affected by humidity. Figure 6 reveals that the oxygen scavenging slowly increases with the relative humidity increase from 32 to 75%. The *OS* values at RH 32% and RH 56% are close to the unactuated film in Figure 6, and the *OS* value is only 0.0027 mol/m^2^ even at RH 75% which is much higher than the relative humidity of common environment. Then, the oxygen scavenging sharply leap to 0.0098 mol/m^2^ at RH 84%. This increase of 263% comparing to the *OS* value at RH 75% implies a drastically oxygen scavenging improvement of the prepared film under extremely high humidity. Finally, *OS* value continuously increase to 0.0152 mol/m^2^ at RH 100%, consisting with the maximal *OS* value of actuated film.

The embedding and release of object molecule in β-cyclodextrin is manly impelled by the water molecule [35,36]. During the embedding process, the water molecule inside β-cyclodextrin is replaced by tea polyphenols which has a higher hydrophobicity than water molecule. However, this replacement is a dynamically balanced and reversible process. Water molecule with high enthalpy (such vapor) can replace the position of tea polyphenols and occupy the cavity of β-cyclodextrin again under high relative humidity, and the tea polyphenols is released as a dissociative state. Meanwhile, LDPE as a typical hydrophobic polymer provides a good vapor barrier and decreases the concentration of water molecule with high enthalpy inside matrix. When the relative humidity is below 75%, the cavity of β-cyclodextrin is mainly occupied by tea polyphenols, and the oxygen scavenging is also restrained. Oppositely, the cavity of β-cyclodextrin is dominantly occupied by water molecule with high enthalpy when relative humidity is up to 84%, and the dissociative tea polyphenols can efficiently absorb oxygen from environment. Thus, it can be observed a drastically oxygen scavenging improvement at RH 84%. The similar result is also obtained in Zhai’s research [37]. This humidity sensibility provides an expectable storage stability under regular storage condition when the film is unused, while the film presents a good oxygen scavenging performance after packaging moist products.

#### 3.3.3. Influence of Activating Condition

**Figure 7:**
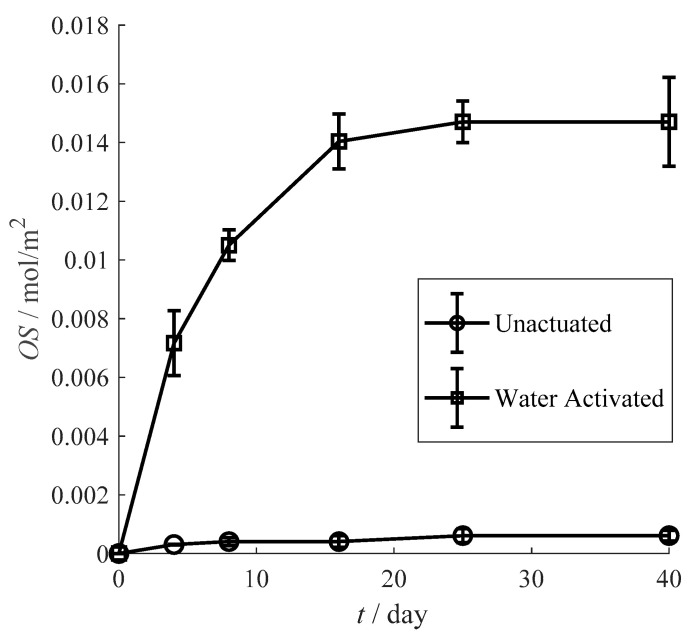
Influence of activating condition on the oxygen scavenging performance.

For the same reason of the oxygen scavenging improvement under high relative humidity, the tea polyphenols inside β-cyclodextrin will be replaced by water molecule with high enthalpy when the inclusion complex contacts with water, then, the tea polyphenols will more effectively absorb oxygen. As description of Figure 7, the oxygen scavenging of unactuated film is only 0.0006 mol/m^2^, while the actuated film is almost 25 times comparing to the unactuated one. Thus, the oxygen scavenging performance of the film shows a significant improvement after actuated by water.

#### 3.3.4. Influence of Temperature

**Figure 8:**
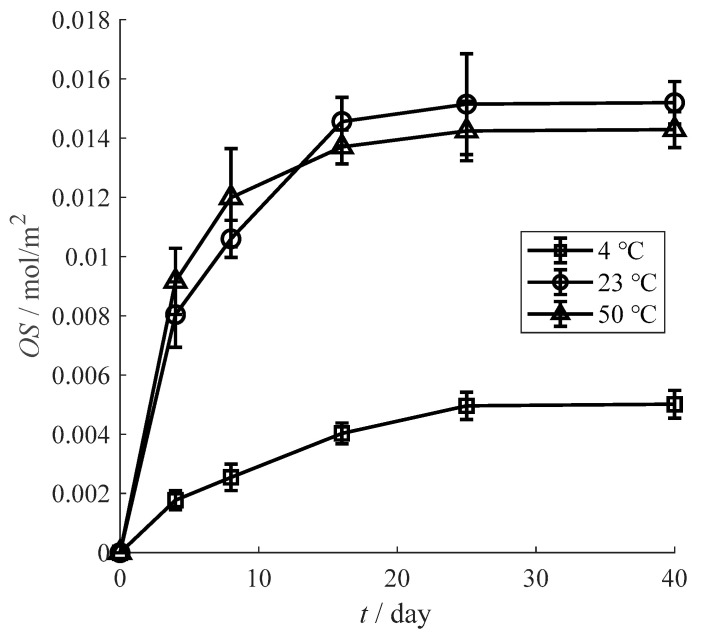
Influence of temperature on the oxygen scavenging performance.

Figure 8 shows the influence of temperature on the oxygen scavenging performance of prepared film. The oxygen scavenging ability of the film is sharply reduce under low temperature of 4 °C due to the reaction activity inhibition of tea polyphenols under low temperature [38]. The reaction activity of tea polyphenols is enhancive with the temperature increase. However, the amount of oxygen scavenging is only determined by the tea polyphenols contents. Thus, the amount of oxygen scavenging at 23 °C is approximate with the result at 50 °C while the oxygen scavenging rate under 50 °C is slightly higher than the rate of 23 °C. This result is consistent with Kelly’s research about gallic acid [39].

### 3.4. Tensile Properties

**Figure 9:**
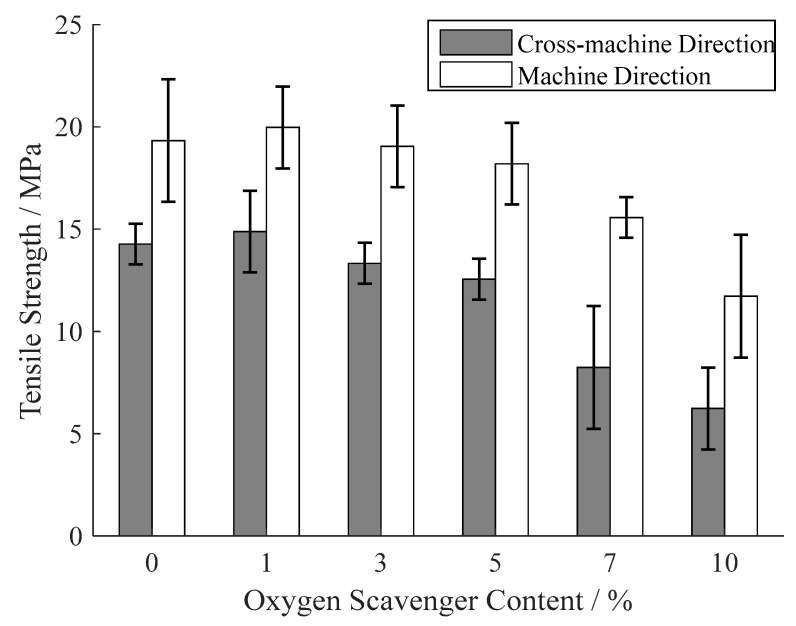
Tensile strength of the prepared films under different oxygen scavenger content.

**Figure 10:**
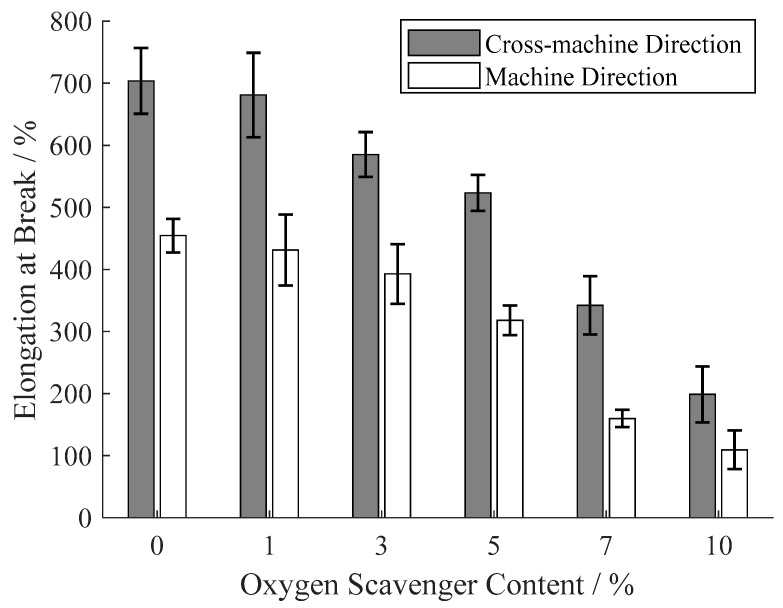
Elongation at break of the prepared films under different oxygen scavenger content.

As the analysis of dispersion in Section 3.2, the tea polyphenols–β-cyclodextrin inclusion complex leads to a heterogeneous dispersion in film base, and this heterogeneity causes a phase separation [40] and stress concentration [41]. Therefore, both of the tensile strengths on cross-machine direction and machine direction reduce with the increase of oxygen scavenger content in Figure 9. This decrease becomes more evident when the oxygen scavenger content is more than 5% due to the more significant heterogeneity at high oxygen scavenger content. Analogously, as shown in Figure 10, the elongation at break of the prepared films also decreases with the oxygen scavenger content increase. However, the tensile strength under oxygen scavenger content of 5% also satisfies the demand of regular food packages [42]. It also can be observed that the tensile strength on the cross-machine direction is significantly weaker than the machine direction, while the elongation at break on the cross-machine direction is higher than the machine direction. This mechanical anisotropy is caused by the molecular orientation during the co-extrusion casting on machine direction.

### 3.5. Heat-Sealing Strength

**Figure 11:**
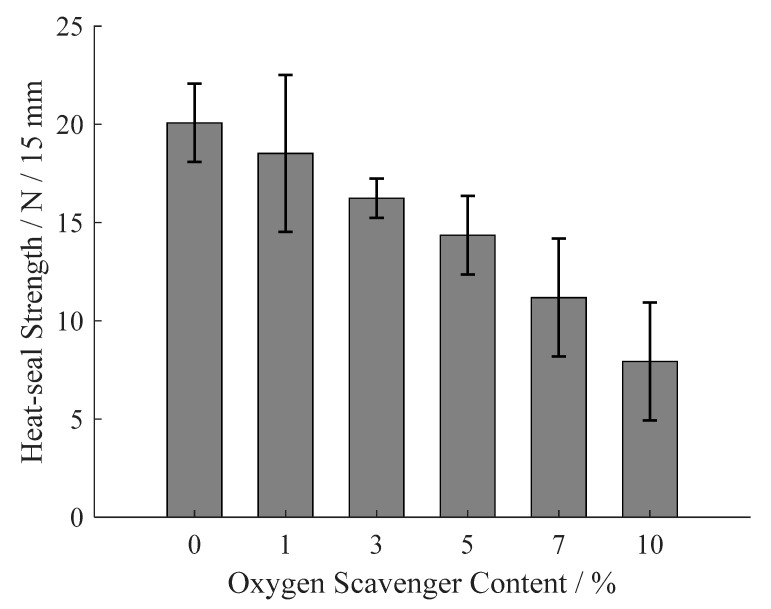
Heat sealing strength of the prepared films under different oxygen scavenger content.

Heat-sealing property is an indispensable performance for food packaging film. As shown in Figure 11, the heat-sealing strength monotonically declines with the oxygen scavenger content increase. The main reason of this deterioration of heat-sealing property is that the rigid oxygen scavenger increases the molecular movement resistance of LDPE during heat-sealing, and impedes the molecular blending on the heat-sealing surface. Thus, the bonding strength between two pieces of film is decrease, and the heat-sealing property is accordingly deteriorative. Although the heat-sealing strength is deteriorated with the adding of oxygen scavenger, it is acceptable comparing to the previous studies [43] at oxygen scavenger content less than 5%.

### 3.6. Application

#### 3.6.1. VC Content

**Figure 12:**
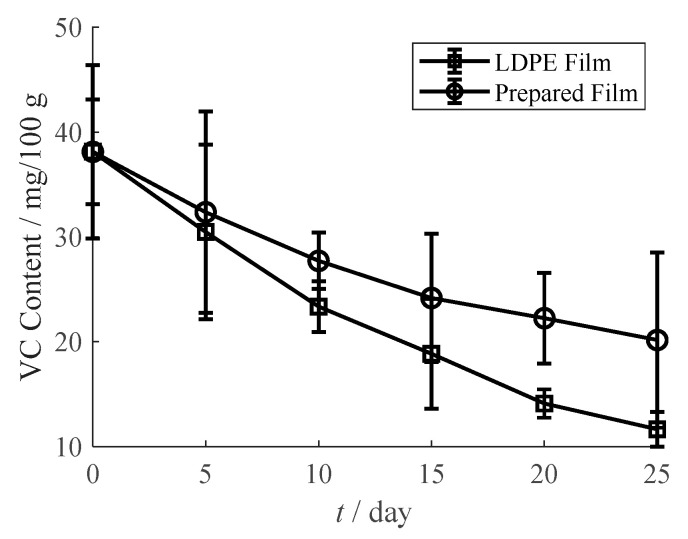
VC content of orange juice in LDPE film pouch and prepared film pouch.

Figure 12 is the VC content change of orange juice in the regular LDPE film pouch and the prepared film pouch. The VC content reduces from 38.12 mg/100 g to 11.65 mg/100 g in the pouch of regular LDPE, while the VC content is 20.17 mg/100 g in the prepared oxygen scavenging film after 25 days storage. This advantage in maintaining VC indicates that the prepared film retards the oxidation of VC in orange juice.

#### 3.6.2. Brown Stain

**Figure 13:**
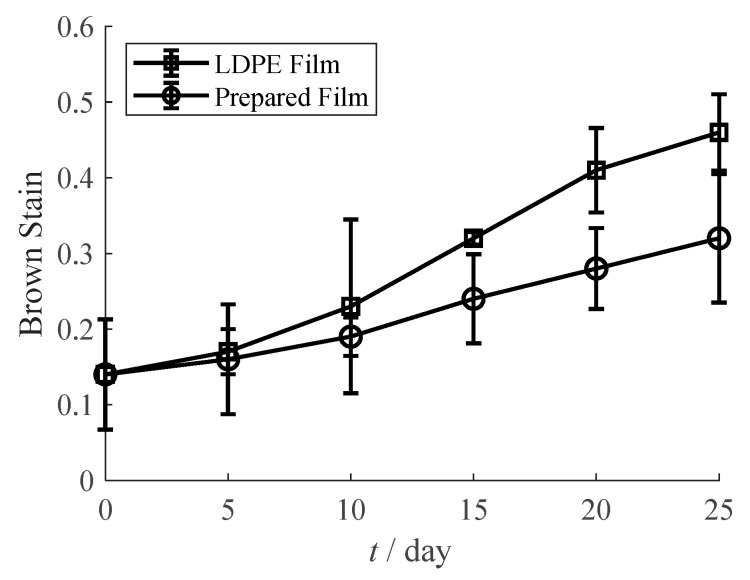
Brown stain of orange juice in LDPE film pouch and prepared film pouch.

The oxidation of orange juice also accelerates both enzymatic and non-enzymatic browning. It can be observed in Figure 13 the brown stain of orange juice in different packages. The initial brown stain of orange juice is only 0.14, and the brown stain significantly increases during storage. For the prepared oxygen scavenging pouch, the brown stain increases to 0.32 after 25 days, while this value has increased to 0.46 in regular LDPE pouch. This result shows a good browning resistance of the prepared oxygen scavenging film.

However, both of the VC content and the brown stain are obvious change comparing the initial state even under the protection of oxygen scavenging film. This is mainly due to that the oxidation process is synergistically affected by oxygen, light, pH value, enzyme and other factors [44]. Thus, a collaboration application of oxygen scavenging films, antiglare materials and enzyme inactivation technology is a more effective method to protect oxygen-sensitive food.

## 4. Conclusions

A moisture activating oxygen scavenging film based on tea polyphenols–β-cyclodextrin inclusion complex was prepared through co-extruding with LDPE. The inclusion structure of tea polyphenols in β-cyclodextrin was proved though Fourier transform infrared spectra, and the oxygen scavenging and mechanical performance were discussed. The results indicate that the prepared film has a relatively homogeneous dispersion of oxygen scavenger when the oxygen scavenger content is less than 5%. However, the dispersion becomes heterogeneous with the oxygen scavenger content increasing from 7 to 10%. The maximal oxygen scavenging is 0.0150 mol/m^2^ in the prepared film with 5% oxygen scavenger, and the amount of oxygen scavenging significantly decreases in the film with higher oxygen scavenger content of 7% and 10%. Moreover, the oxygen scavenging amount sharply increase after steeping in water or storage in extremely high humidity of RH 84% while the oxygen scavenging is restrained under RH 32–75%. This obvious moisture activating and humidity sensitivity is advantageous for the stability during film storage and the effectiveness for film application. Furthermore, both of the tensile strength and heat-sealing strength residuals of the prepared film with 5% oxygen scavenger are acceptable, and these two performances sharply decline at 7% and 10% oxygen scavenger. Finally, an application of this oxygen scavenging film used in orange juice is implemented, and the results shows a good antioxidant performance of the prepared film. Thus, the optimal oxygen scavenger content of this film is 5%, and this acquired moisture activating oxygen scavenging film can be potentially applied for protection of oxygen-sensitive food with high moisture content.

## Figures and Tables

**Table 1:** Component of the oxygen scavenging film.

LDPE Resin/g	Oxygen Scavenger/g	Oxygen Scavenger Content/%
500	0	0
455	5	1
485	15	3
475	25	5
465	35	7
450	50	10

**Table 2:** Parameters of co-extrusion casting machine.

Feeding Section	Compression Section	Homogenizing Section	Cast Die Section	Feeding Velocity	Screw Velocity	Draw Down Ratio (Machine Direction)
160 °C	165 °C	170 °C	170 °C	5 kg/h	15 rpm	7.8

**Table 3:** Relative humidity of saturated solution at 30 °C [30].

Saturated Solution	MgCl_2_	NaBr	NaCl	KCl	K_2_SO_4_	H_2_O
RH/%	32.44 ± 0.14	56.03 ± 0.38	75.09 ± 0.11	83.62 ± 0.25	97.00 ± 0.40	100.00
